# Supplementary material for: Identification of BRCA1/2 mutation female carriers using circulating microRNA profiles
Source: Nat Commun. 2023 Jun 8;14:3350. doi: 10.1038/s41467-023-38925-4 (PMC10250543; doi:10.1038/s41467-023-38925-4)
Supplement: Supplementary file 1 — Supplementary Information [file 41467_2023_38925_MOESM1_ESM.pdf]

Supplementary Figures:

Supplementary Figure 1 - PCA representation of samples from all evaluated cohorts A) without batch adjustment; B) after batch adjustment with UPenn cohort as unmodified reference.

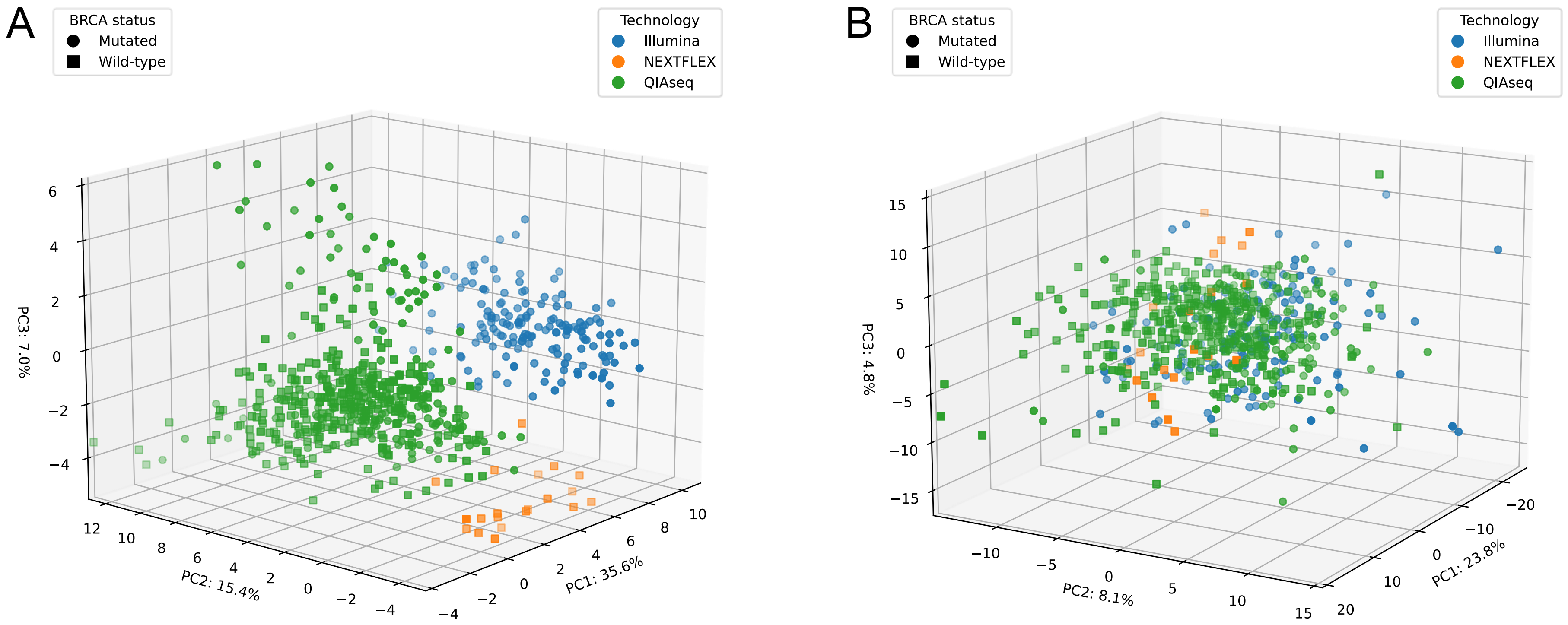

Supplementary Figure 2 – Heatmap of expression values of miRNAs used in the classifcation model in the UPenn group

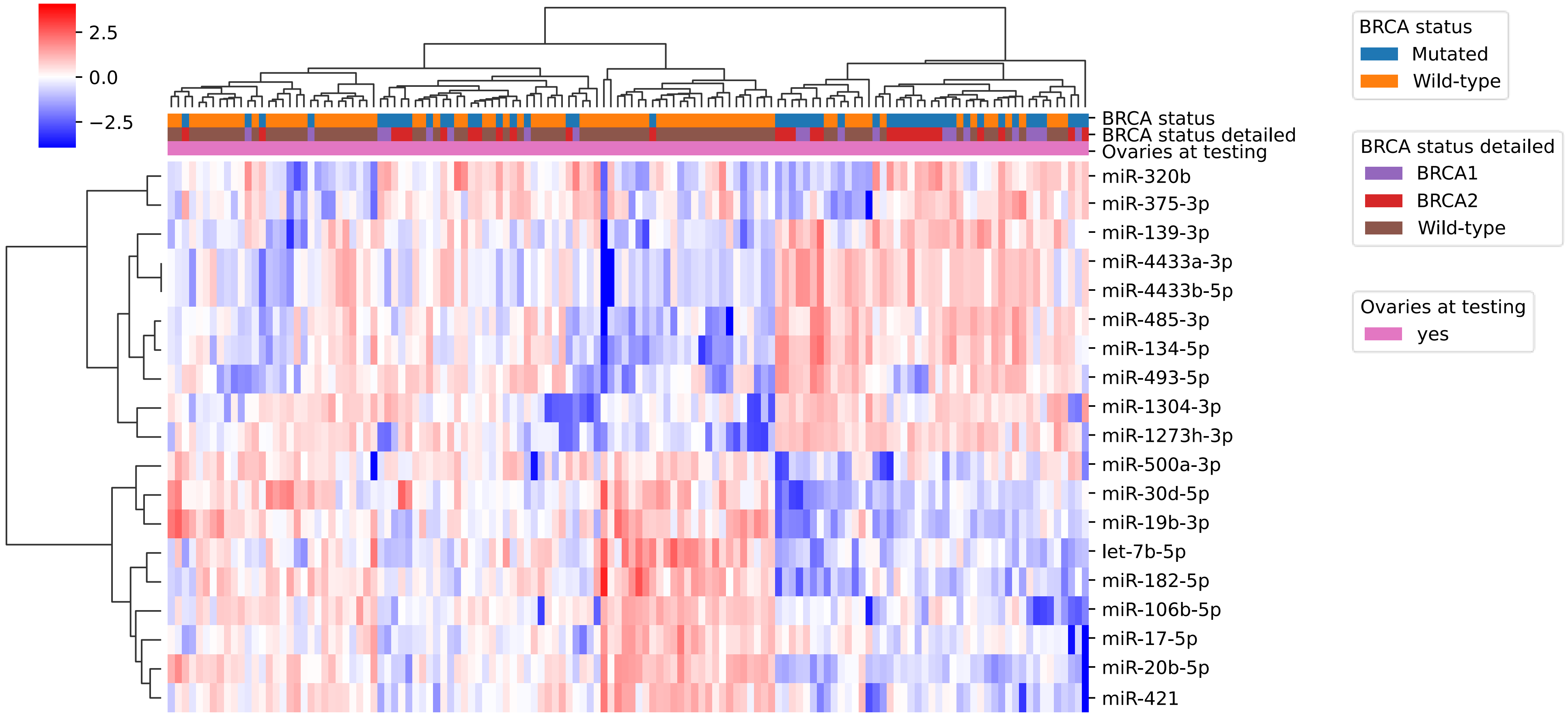

Supplementary figure 3 - Positive (A) and negative (B) predictive values vs prevalence of mutations in homologous recombination genes in population. The corresponding sensitivity and specificity values for different two-sided probability cutoffs are, respectively: 97.1% and 37.6% for p=0.1, 94.3% and 58.1% for p=0.25, 86% and 80.5% for p=0.5, 64.6% and 94.7% for p=0.75, 34.9% and 99.3% for p=0.9.

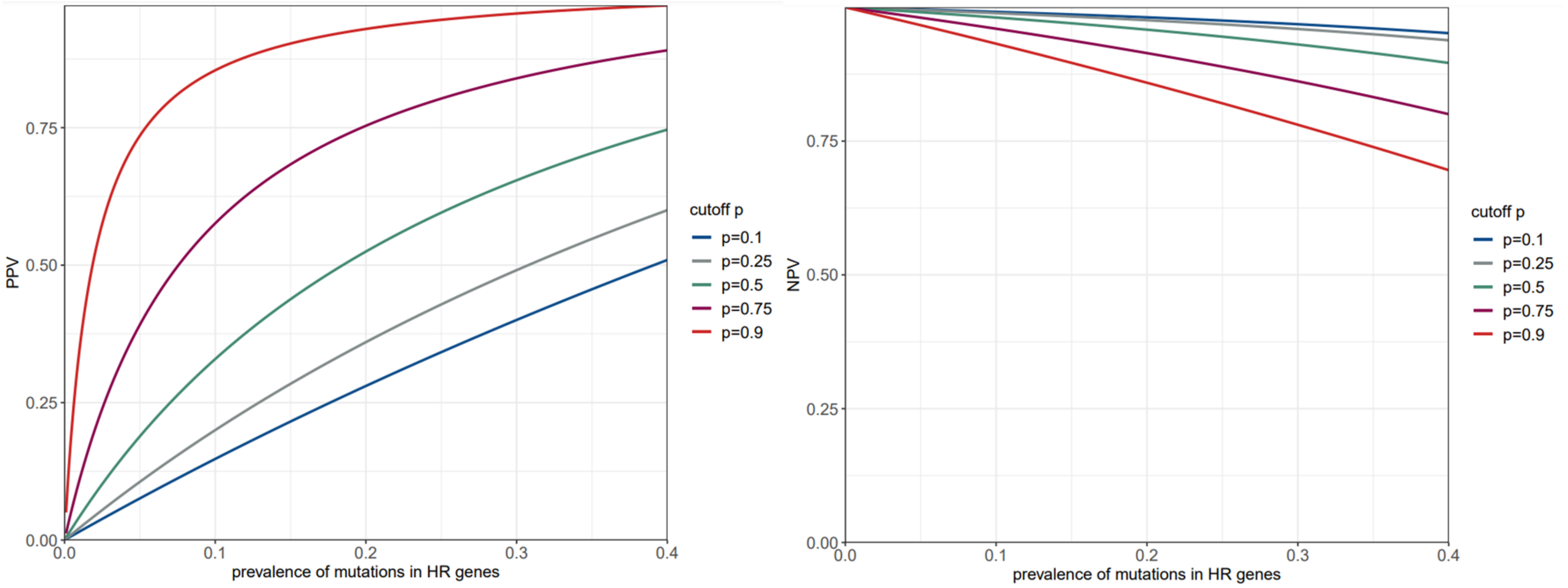

Supplementary figure 4 - Estimated probabilities of BRCAmt for patients with known age depending on the presence of pathogenic mutations of *BRCA1* or *BRCA2*.

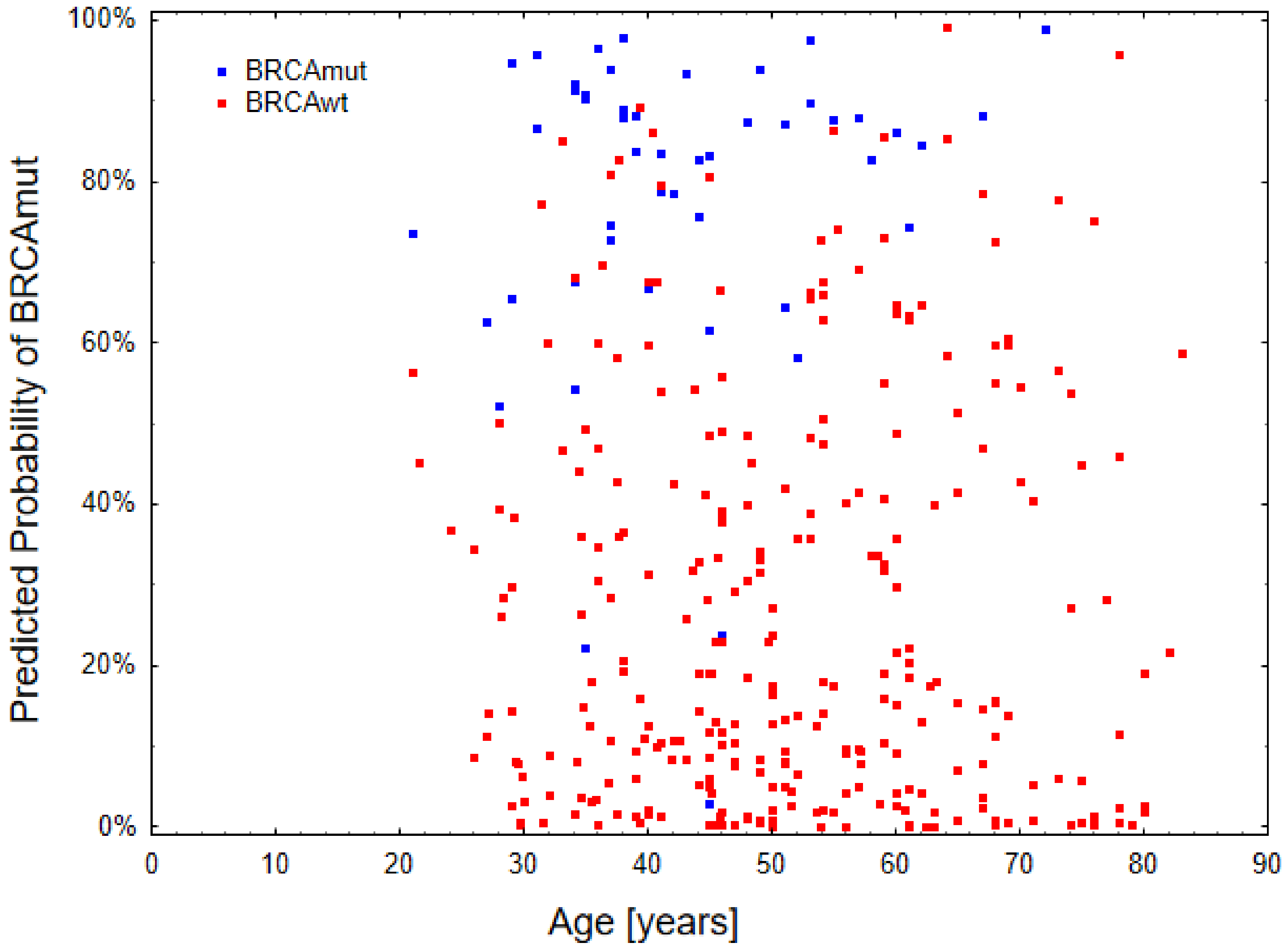

Supplementary Tables:

Supplementary Table 1. Clinical characteristics of all cohorts. BWH - Brigham and Women's Hospital; CCGP - Center for Cancer Genetics and Prevention at DFCI; DGO - Department of Gynaecological Oncology, Tata Medical Center, Kolkata, India; IHCC - International Hereditary Cancer Center of the Pomeranian Medical University, Poland; DFCI - DFCI/BWH biobank; UPenn - University of Pennsylvania.

Variable	Level	BWH (N=87)	CCGP (N=162)	DGO (N=20)	IHCC (N=52)	DFCI (N=200)	UPenn (N=132)
Menopausal status	Post-menopausal	0	0	4 (20.0%)	-	119 (59.5%)	31 (23.5%)
	Pre-menopausal	0	0	5 (25.0%)	-	81 (40.5%)	101 (76.5%)
	Unknown	87 (100%)	162 (100%)	11 (55.0%)	52 (100%)	0	0
Age [years]	Median (25%-75%), Min-Max	-	-	40.5 (29.0-58.8), 28.0-71.0	-	54.0 (46.0-65.0), 24.0-83.0	40.5 (35.0-49.0), 21.0-72.0
	No data	87 (100%)	162 (100%)	12 (60%)	52 (100%)	0	0
Ca-125	Median (25%-75%), Min-Max	-	-	173.6 (21.7-328.4), 8.6-2889.0	-	15.0 (11.0-31.0), 0.0-2427.0	12.0 (9.0-16.0), 7.0-88.0
	No data	87 (100%)	162 (100%)	14 (70%)	52 (100%)	118 (59.0%)	119 (90.2%)
Having ovaries at testing	Yes	40 (46%)	134 (82.7%)	20 (100%)	52 (100%)	200 (100%)	132 (100%)
	No	47 (54%)	28 (17.3%)	0	0	0	0

Supplementary Table 2 - Logistic regression model parameters used for predicting the BRCA status. Estimates, odds ratios and two-sided p values were estimated on the training set of samples. The final model with a cut-off value of 50% probability for declaring positive calls was evaluated on the validation cohort.

	Univariable analysis				Multivariable analysis				
miRNAs	OR	(-95%CI)	(+95%CI)	p value	Estimate	OR	(-95%CI)	(+95%CI)	p value
Intercept					34.23				
hsa.miR.20b.5p	0.26	0.20	0.33	<0.001	-0.21	0.81	0.55	1.17	0.300
hsa.miR.19b.3p	0.19	0.14	0.26	<0.001	-0.81	0.44	0.27	0.71	<0.001
hsa.let.7b.5p	0.11	0.07	0.16	<0.001	-2.05	0.13	0.07	0.24	<0.001
hsa.miR.320b	3.81	2.90	5.11	<0.001	0.94	2.55	1.70	3.92	<0.001
hsa.miR.139.3p	3.16	2.46	4.11	<0.001	0.23	1.26	0.87	1.82	0.200
hsa.miR.30d.5p	0.19	0.13	0.27	<0.001	-0.90	0.41	0.24	0.67	<0.001
hsa.miR.17.5p	0.32	0.25	0.41	<0.001	-0.04	0.96	0.63	1.44	0.800
hsa.miR.182.5p	0.37	0.30	0.46	<0.001	0.39	1.48	1.03	2.16	0.036
hsa.miR.421	0.37	0.28	0.49	<0.001	0.30	1.36	0.95	1.95	0.093
hsa.miR.375.3p	1.64	1.44	1.87	<0.001	0.27	1.31	1.09	1.58	0.004

Supplementary Table 3 - Confusion matrix of the final classification model.

		BRCAwt	BRCAmut	Sensitivity	Specificity	PPV	NPV
Training set	Predicted BRCAwt	131	35	84.51%	79.39%	84.89%	78.92%
	Predicted BRCAmut	34	191				
Testing set	Predicted BRCAwt	46	11	85.33%	83.64%	87.67%	80.70%
	Predicted BRCAmut	9	64				
Validation set	Predicted BRCAwt	67	3	93.88%	80.72%	74.19%	95.71%
	Predicted BRCAmut	16	46				

Supplementary Table 4 - Performance of the test for detecting *BRCA1* or *BRCA2* mutations depending on menopausal status of the tested patients.

			Actual class					
	Menopausal status	Predicted class	Mutated	Wild-type	Total	Sensitivity	Specificity	Accuracy
N	Unknown	BRCAmut	180	5	185	80%	94%	84%
%			79.65%	5.81%	56.40%			
N		BRCAwt	46	81	127			
%			20.35%	94.19%	39.08%			
N	Postmenopausal	BRCAmut	42	20	62	84%	81%	82%
%			84.00%	19.23%	18.90%			
N		BRCAwt	8	84	92			
%			16.00%	80.77%	28.31%			
N	Premenopausal	BRCAmut	64	17	81	86%	85%	86%
%			86.49%	15.04%	24.70%			
N		BRCAwt	10	96	106			
%			13.51%	84.96%	32.62%			
Grand total			350	303	653			

Supplementary Table 5 - Performance of the classification model depending on the patients' age by decade.

			Actual class					
	Age category	Predicted class	BRCAmut	BRCAwt	Total	Sensitivity	Specificity	Accuracy
N	<30	BRCAmut	1	0	1	100%	100%	100%
%			100.00%	0.00%				
N		BRCAwt	0	3	3			
%			0.00%	100.00%				
N	30-39	BRCAmut	8	4	12	80%	88%	86%
%			80.00%	11.76%				
N		BRCAwt	2	30	32			
%			20.00%	88.24%				
N	40-49	BRCAmut	18	10	28	95%	82%	85%
%			94.74%	17.86%				
N		BRCAwt	1	46	47			
%			5.26%	82.14%				
N	50-59	BRCAmut	8	8	16	73%	91%	89%
%			72.73%	9.30%				
N		BRCAwt	3	78	81			
%			27.27%	90.70%				
N	60-69	BRCAmut	6	12	18	100%	80%	82%
%			100.00%	20.34%				
N		BRCAwt	0	47	47			
%			0.00%	79.66%				
N	70-79	BRCAmut	2	6	8	100%	83%	84%
%			100.00%	17.14%				
N		BRCAwt	0	29	29			
%			0.00%	82.86%				
N	80+	BRCAmut	0	2	2	NA	89%	89%
%			NA	11.11%				
N		BRCAwt	0	16	16			
%			NA	88.89%				
N	Unknown	BRCAmut	243.00	0.00	243	81%	100%	81%
%			80.73%	0.00%				
N		BRCAwt	58.00	12.00	70			
%			19.27%	100.00%	21.54%			
Grand total			49	291	340			
