# Supplementary material for: Identification of BRCA1/2 mutation female carriers using circulating microRNA profiles
Source: Nat Commun. 2023 Jun 8;14:3350. doi: 10.1038/s41467-023-38925-4 (PMC10250543; doi:10.1038/s41467-023-38925-4)
Supplement: Supplementary file 4 — Description of Additional Supplementary Files [file 41467_2023_38925_MOESM4_ESM.docx]

**Inventory of Supplementary Information**

Supplementary Figure 1 - PCA representation of samples from all evaluated cohorts A) without batch adjustment; B) after batch adjustment with UPenn cohort as unmodified reference.

Supplementary Figure 2 – Heatmap of expression values of miRNAs with convergent *BRCA*-mt; *BRCA*-wt profiles regardless of data preprocessing in the UPenn group.

Supplementary Figure 3 - Positive (A) and negative (B) predictive values vs prevalence of mutations in homologous recombination genes in population. The corresponding sensitivity and specificity values for different probability cut-offs are, respectively: 97.1% and 37.6% for p=0.1, 94.3% and 58.1% for p=0.25, 86% and 80.5% for p=0.5, 64.6% and 94.7% for p=0.75, 34.9% and 99.3% for p=0.9.

Supplementary Figure 4 - Estimated probabilities of *BRCA*-mt for patients with known age depending on the presence of pathogenic mutations of *BRCA1* or *BRCA2*.

Supplementary Table 1. Clinical characteristics of all cohorts. BWH - Brigham and Women's Hospital; CCGP - Center for Cancer Genetics and Prevention at DFCI; DGO - Department of Gynaecological Oncology, Tata Medical Center, Kolkata, India; IHCC - International Hereditary Cancer Center of the Pomeranian Medical University, Poland; DFCI - DFCI/BWH biobank; UPenn - University of Pennsylvania.

Supplementary Table 2 - Logistic regression model parameters used for predicting the BRCA status. Estimates, odds ratios and two-sided p values were estimated on the training set of samples. The final model with a cut-off value of 50% probability for declaring positive calls was evaluated on the validation cohort.

Supplementary Table 3 - Confusion matrix of the final classification model.

Supplementary Table 4 - Performance of the test for detecting *BRCA1* or *BRCA2* mutations depending on menopausal status of the tested patients.

Supplementary Table 5 - Performance of the classification model depending on the patients' age by decade.

Supplementary Dataset 1 - Full results of differential expression analysis on unadjusted data.

Supplementary Dataset 2 - Full results of differential expression analysis on batch-adjusted data.

Supplementary Dataset 3 - Variable selection procedures used to identify a subset of miRNAs with the best class separation properties.

Supplementary Dataset 4 - Classification models deployed on the dataset to identify a model with the best diagnostic performance. Training accuracy and AUC ROC metrics in the case of methods utilizing balanced datasets (with SMOTE) were provided as for balanced training datasets. Abbreviations of selected methods correspond to OmicSelector documentation (<https://kstawiski.github.io/OmicSelector/articles/metody.html>).

Supplementary Dataset 5 - Sample-level data of all cohorts included in the study. BWH - Brigham and Women's Hospital; CCGP - Center for Cancer Genetics and Prevention at DFCI; DGO - Department of Gynaecological Oncology, Tata Medical Center, Kolkata, India; IHCC - International Hereditary Cancer Center of the Pomeranian Medical University, Poland; DFCI - DFCI/BWH biobank; UPenn - University of Pennsylvania.

Supplementary Dataset 6 - Sample-level data of all cohorts included in the study with class predictions for the final model. Expression values are presented as log2(TPM). BWH - Brigham and Women's Hospital; CCGP - Center for Cancer Genetics and Prevention at DFCI; DGO - Department of Gynaecological Oncology, Tata Medical Center, Kolkata, India; FCCC – Fox Chase Cancer Center, IHCC - International Hereditary Cancer Center of the Pomeranian Medical University, Poland; DFCI - DFCI/BWH biobank; UPenn - University of Pennsylvania.

Supplementary Dataset 7 - Raw miRNA counts (unprocessed) of all samples

Supplementary Dataset 8 - Raw miRNA counts (log2(TPM)) of all samples.

Supplementary Dataset 9 - Batch adjusted miRNA counts (log2(TPM)) of all samples.

Supplementary Code 1 - Model discriminating between *BRCA*-mt and *BRCA*-wt controls from the UPenn dataset. The model can be used for scoring via OmicSelector software or using caret R package. Model can be loaded using basic readRDS() function and scored using predict() function. Supplementary Table 8 can be used as input file for model scoring.
